# Supplementary material for: On-demand ride service platform with differentiated services
Source: PLoS One. 2024 Jan 12;19(1):e0296732. doi: 10.1371/journal.pone.0296732 (PMC10786389; doi:10.1371/journal.pone.0296732)
Supplement: Attachment — Submitted filename: 2023_04_17_Comments_On-demand ride service platform with differentiated services.docx [file pone.0296732.s001.docx]

**On-demand ride service platform with differentiated services**

This paper explores the pricing strategy considering the influence of consumer preference heterogeneity and different service types of drivers with network externalities. The research content is relatively complete and the writing is standardized; however, the paper still needs **minor revision** before it is accepted and published. My detailed comments are as follows:

1. Some of the expressions in the text are too repetitive, and it is suggested to make appropriate deletions to make the writing clearer and increase readability.
2. The assumptions of the model are too idealistic, and the driver's income equation considers limited factors. The income is also affected by factors such as demand time, and distance etc., which lack corresponding literature support.
3. The parameter settings in the numerical analysis section lack necessary practical support and corresponding data sources, which reduces the realism of the paper.
4. The first and third conclusions, as well as the second and fourth conclusions, are quite similar and could be merged. It is recommended to delete overly intuitive conclusions and add more managerial insights.
5. Additionally, I recommend that the authors consider delegating the writing to a professional linguistic agency to improve the language expression.
